# Seroprevalence and risk factors of SARS-CoV-2 infection in an urban informal settlement in Nairobi, Kenya, December 2020

**DOI:** 10.12688/f1000research.72914.1

**Published:** 2021-08-26

**Authors:** Patrick K Munywoki, Caroline Nasimiyu, Moshe Dayan Alando, Nancy Otieno, Cynthia Ombok, Ruth Njoroge, Gilbert Kikwai, Dennis Odhiambo,, Mike Powel Osita, Alice Ouma, Clifford Odour, Bonventure Juma, Caroline A Ochieng, Immaculate Mutisya, Isaac Ngere, Jeanette Dawa, Eric Osoro, M Kariuki Njenga, Godfrey Bigogo, Peninah Munyua, Terrence Q Lo, Elizabeth Hunsperger, Amy Herman-Roloff

**Affiliations:** 1 Center for Global Health, Division of Public Health Protection, U.S. Centers for Disease Control and Prevention, Nairobi, USA; 2 Global Health Kenya, Washington State University, Nairobi, USA; 3 Paul G. Allen School of Global Health, Washington State University, Pullman, USA; 4 Centre for Global Health Research, Kenya Medical Research Institute (KEMRI), Nairobi, Kenya; 5 Centre for Global Health Research,, Kenya Medical Research Institute, Kisumu, Kenya

**Keywords:** Population-based, Households, Serosurvey, Serology, IgM and IgG, SARS-CoV-2, COVID-19, urban informal settlement, Kibera, Kenya

## Abstract

**Introduction: **Urban informal settlements may be disproportionately affected by the COVID-19 pandemic due to overcrowding and other socioeconomic challenges that make adoption and implementation of public health mitigation measures difficult. We conducted a seroprevalence survey in the Kibera informal settlement, Nairobi, Kenya, to determine the extent of SARS-CoV-2 infection.

**Methods: **Members of randomly selected households from an existing population-based infectious disease surveillance (PBIDS) provided blood specimens between 27 ^th^ November and 5 ^th^ December 2020. The specimens were tested for antibodies to the SARS-CoV-2 spike protein. Seroprevalence estimates were weighted by age and sex distribution of the PBIDS population and accounted for household clustering. Multivariable logistic regression was used to identify risk factors for individual seropositivity.

**Results: **Consent was obtained from 523 individuals in 175 households, yielding 511 serum specimens that were tested. The overall weighted seroprevalence was 43.3% (95% CI, 37.4 – 49.5%) and did not vary by sex. Of the sampled households, 122(69.7%) had at least one seropositive individual. The individual seroprevalence increased by age from 7.6% (95% CI, 2.4 – 21.3%) among children (<5 years), 32.7% (95% CI, 22.9 – 44.4%) among children 5 – 9 years, 41.8% (95% CI, 33.0 – 51.1%) for those 10-19 years, and 54.9%(46.2 – 63.3%) for adults (≥20 years). Relative to those from medium-sized households (3 and 4 individuals), participants from large (≥5 persons) households had significantly increased odds of being seropositive, aOR, 1.98(95% CI, 1.17 – 1.58), while those from small-sized households (≤2 individuals) had increased odds but not statistically significant, aOR, 2.31 (95% CI, 0.93 – 5.74).

**Conclusion: **In densely populated urban settings, close to half of the individuals had an infection to SARS-CoV-2 after eight months of the COVID-19 pandemic in Kenya. This highlights the importance to prioritize mitigation measures, including COVID-19 vaccine distribution, in the crowded, low socioeconomic settings.

## Introduction

Recent discovery and spread of the Severe Acute Respiratory Syndrome Coronavirus 2 (SARS-CoV-2), and the resulting disease associated with this virus, Coronavirus Disease 2019 (COVID-19), has brought unprecedented morbidity and mortality worldwide. ^1- 3^ Tracking the extent of the virus spread and disease severity in various populations is important in informing the local, national, and global public health response. Real time reverse transcription–polymerase chain reaction (rRT-PCR) testing has been the mainstay diagnostic test for COVID-19 surveillance. rRT-PCR is expensive and requires specialised infrastructure, equipment, and skills. These laboratory challenges compounded by global shortages of supplies and restrictions in shipping has resulted in sub-optimal implementation of rRT-PCR in low resource settings. Serologic tests that are cheaper than rRT-PCR are important in determining population level prevalence of SARS-CoV-2 infections. Infected individuals, including those with asymptomatic and mild disease, develop an immune response with detectable antibodies within two weeks of exposure ^4, 5^ and for months afterwards ^6^ allowing inferences to be made on the true extent of exposure in the population.

In Kenya, the first case of SARS-CoV-2 infection was detected on 12 ^th^ March 2020, and as of 30 ^th^ November 2020, a total of 83,316 rRT-PCR confirmed cases and 1,452 deaths (case fatality rate, 1.7%) were reported by the Ministry of Health (MoH). ^7, 8^ The national MoH data shows two major waves of increased transmission in Kenya observed prior to this serosurvey; the first wave happened between June and August 2020 and the second wave between October and November 2020. ^9^ Nevertheless, with limited testing resources, Kenya implemented a strategy to prioritize testing only symptomatic persons who presented at health facilities and met the suspect case definitions. ^10^ Along with the suboptimal contact tracing, the MoH's counts likely underreports cases by excluding individuals with asymptomatic and mild cases of COVID-19 who are less likely to seek healthcare. Serologic testing may offer additional surveillance insights. Previous findings from SARS-CoV-2 antibody testing of serum from Kenya's National Blood Transfusion Services by Kenya Medical Research Institute (KEMRI)-Wellcome Trust investigators correlated well with the observed increase in community transmissions. The investigators reported a marked increase in crude prevalence of SARS-CoV-2 antibodies from 5.6% in May to 13.3% by August 2020. ^11, 12^ In Nairobi County, the increase in seroprevalence was more than double in the same period; from a baseline of 8.9% in May to 21.5% in August 2020.

The distribution of SARS-CoV-2 infections is unlikely to be homogeneous across all communities and regions, and informal settlement environments such as Kibera in Nairobi may be disproportionately affected due to overcrowding, water, sanitation, and hygiene (WASH) infrastructure constraints, and socio-economic challenges that make adoption and implementation of COVID-19 public health mitigation measures difficult. A serosurvey in July 2020 in Mumbai, India found the seroprevalence among slum residents to be nearly 3.6 times that of non-slum residents. ^13^ There have been very few serosurvey data in informal settlements in Kenya. ^14^ This article provides findings on seroprevalence and risk factors associated with history of SARS-CoV-2 infection from a population-based seroprevalence survey in Kibera, the largest urban, densely populated, informal settlement in Nairobi, Kenya.

## Methods

### Study site and population

**Figure 1.**
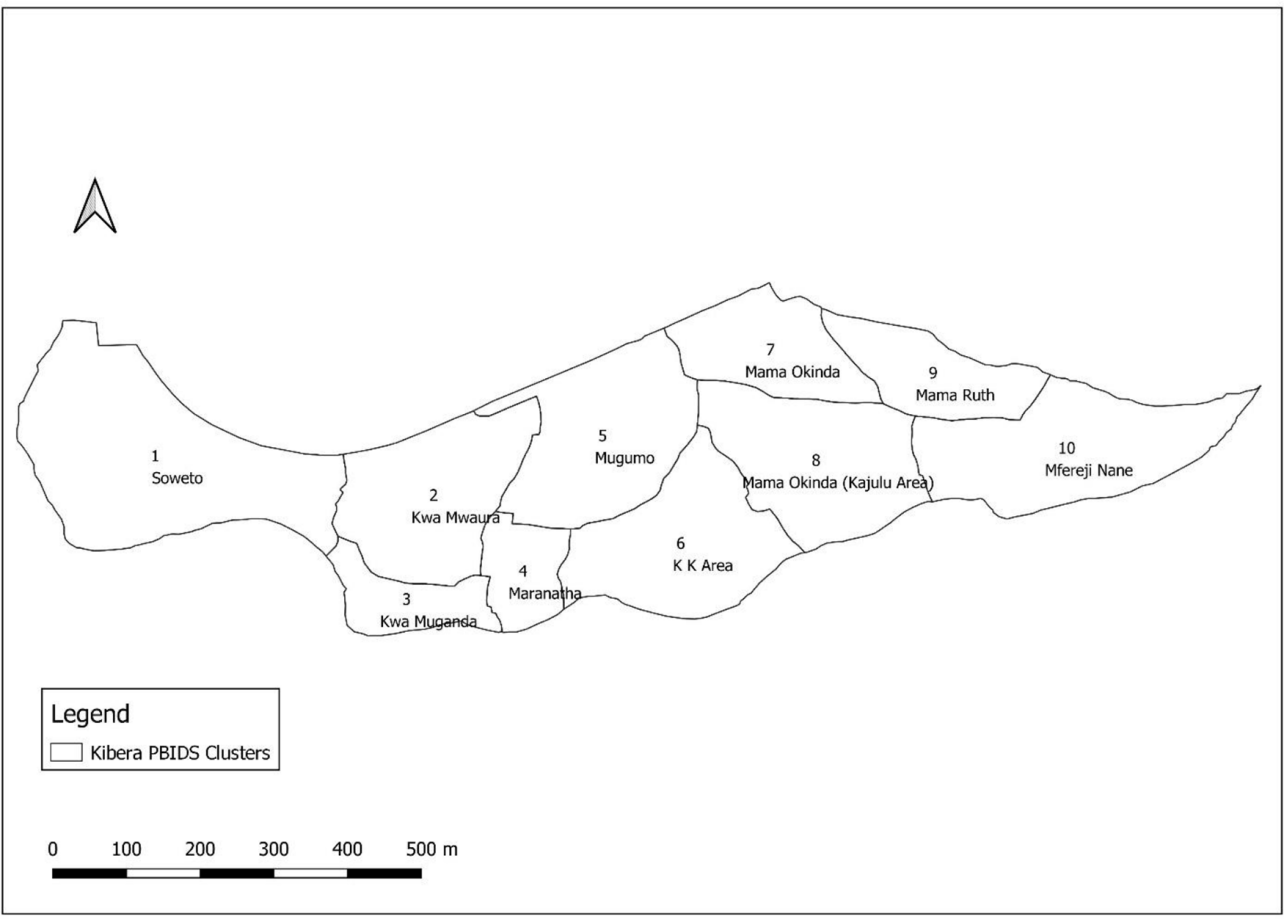
Map showing the area under population-based infectious disease surveillance (PBIDS) in Kibera informal settlement, Nairobi, Kenya. The subdivisions show the areas of residence i.e. PBIDS zones 1-10.

Kibera is a densely populated informal settlement within Nairobi, Kenya, characterised by overcrowding, poor sanitation, and poor infrastructure. This seroprevalence survey was embedded in an ongoing population-based infectious disease surveillance (PBIDS) within the informal settlement. ^15, 16^ The Kibera PBIDS covers an area < 0.5 km ^2^ with an estimated population of about 23,103 individuals in 5,265 households (as of December 2020) living in two villages, Soweto and Gatwekera, that are under active surveillance. The PBIDS area is divided into 10 zones, referred to as residential areas ( Figure 1). The platform is maintained by KEMRI-Centre for Global Health Research (CGHR) and Washington State University-Global Health in Kenya (WSU-GH) with technical and financial support from U.S. Centers for Disease Control and Prevention (CDC). The seroprevalence survey in Kibera was implemented at the same time as a wider seroprevalence survey across Nairobi County. ^17^

### Sample size calculation

We selected a sample size of 684 persons from 171 households (assuming each household had an average of 4 individuals). This was based on an expected seroprevalence of 25% with a precision of 5.0%, a design effect of 2, and 20% attrition should a repeat seroprevalence survey be possible in the future. More households (n = 181) were eventually included to boost the number of participants enrolled.

### Household selection and enrolment

We conducted a cross-sectional household-based survey aligned with World Health Organisation's (WHO) UNITY seroepidemiological protocol. ^18^ The study households were randomly selected from the most recent PBIDS dataset and household members were consented before enrolment. Efforts were made to recruit all household members, both adults and children, regardless of current or prior COVID-19 infection status. Individuals residing in the selected households who were not yet registered in the PBIDS platform were also approached for consenting if they were residents for a minimum of four months. We conducted a minimum of three study visits to a household before replacing it due to unavailability of household members. When a household was enrolled in the study, we conducted a minimum of three return visits for household members not available at the time of the initial study visit. The household enrolment and data collection were conducted from 27 ^th^ November to 5 ^th^ December 2020 by five trained field teams, each consisting of a field worker and a phlebotomist.

### Data and specimen collection

All participants were interviewed for sociodemographic data such as age, sex, education level, and occupation. Data on current occupation and highest education level were collected from adult participants (≥18 years) only. Data were collected and managed using REDCap (Research Electronic Data Capture) electronic data tools hosted at Washington State University. ^19, 20^ Venous blood samples (approximately 5 ml for persons aged >12; 2-3 ml for children 2-12 years and 1.5 ml for children <2 years) were collected from each participant and transported in a cool box at 2-8°C to the KEMRI-Diagnostics and Laboratory Systems Program (DLSP) laboratory in Nairobi the same day. Sera were extracted from the whole blood specimen and stored at −80°C before testing.

### Serological testing

We tested for total immunoglobulins (IgM and IgG) antibodies to the SARS-CoV-2 spike protein using the Wantai SARS-CoV-2 two-step antigen sandwich enzyme immunoassay kit (Catalogue number, WS-1096; Wantai Biological Pharmacy Enterprise Ltd, Beijing, China). The assay was optimised at KEMRI-DLSP serology lab by including 10 washes instead of five washes recommended by the manufacturer to reduce background cross-reactivity, as described elsewhere. ^17^ The test results were considered positive when the ratio of antibody titer in the sample to a negative control exceeded 1.5 according to manufacturer's instructions. All lab tests were performed in an ISO15189 certified and GCP-accredited KEMRI-DLSP laboratory in Nairobi, Kenya.

### Statistical analysis

**Figure 2.**
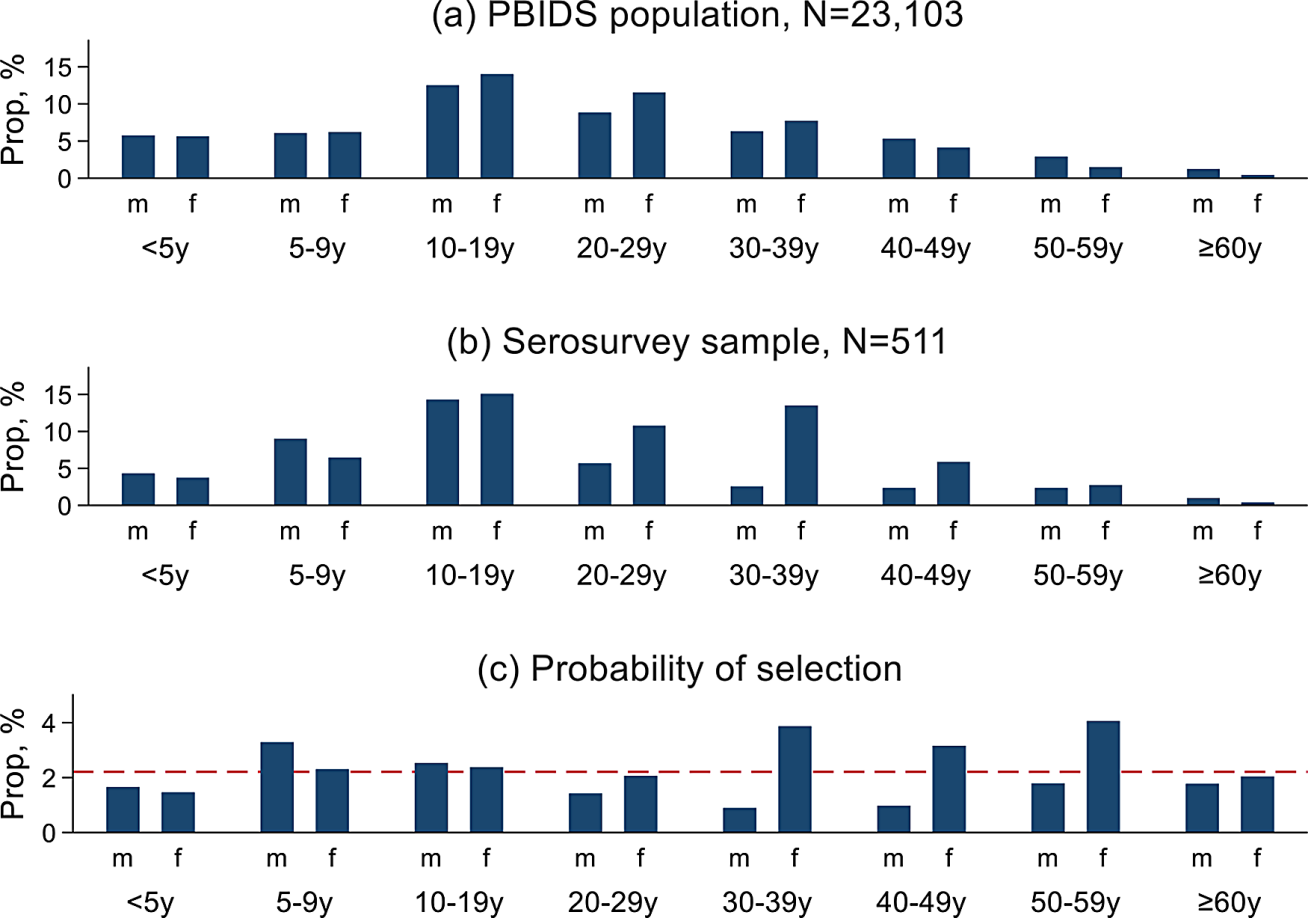
Figure showing age and sex distribution of (a) population-based infectious disease surveillance (PBIDS) population as of December 2020; (b) serosurvey participants; and (c) the probability of participant selection from the PBIDS population into the serosurvey. The red dashed line shows the overall expected probability of selection with bars above the red line indicating overrepresentation while those below the line denoting underrepresentation of the age-sex groups in the sero serosurvey.

Individual seroprevalence of SARS-CoV-2 antibodies was expressed as a percentage of the seropositive among the individuals tested. The disaggregated individual seropositivity estimates accounted for household clustering and weighted by the age and sex structure of the PBIDS population ( Figure 2). The standard errors for generating the 95% confidence intervals were computed using the Taylor linearized variance estimation method. ^21^ Pearson's chi-square test was used to assess the association of categorical variables with individual seropositivity. Household seroprevalence (defined as the percentage of households with at least one seropositive member) was estimated and stratified by household size (usual number of persons living in the household), number of persons enrolled in the serosurvey per household and location in the PBIDS area. Age, sex, area of residence, relationship to head of household, household size, and underlying medical conditions (known hypertensive, asthmatic or diabetic) were considered in the univariable logistic regression model for determining the factors associated with individual seropositivity. Age and sex were considered a priori for inclusion in the multivariable logistic regression. The final multivariable logistic regression model included statistically significant variables, accounting for sampling weights and clustering by household using the clustered sandwich estimator. ^22, 23^ Adjusted odds ratio (aOR) and 95% confidence intervals (CI) were presented and two-sided p-values <0.05 were considered significant.

Stata 15.1 software [STATA Corp, Texas, USA] was used for random selection of households to be enrolled in the study, data cleaning, management, and analyses.

### Ethical considerations

Individual written informed consent/assent was obtained from all the study participants and/or their parents/guardian. Ethical approval for the study was provided by the KEMRI Scientific and Ethical Review Committee in Kenya (#4098) and reliance approval provided by the Washington State University. This activity was also reviewed by CDC and was conducted consistent with applicable federal law and CDC policy as provided for in the Code of Federal Regulations (45 C.F.R part 46 and 21 C.F.R. part 56). The PBIDS platform is approved by KEMRI Scientifical and Ethical Review Committee in Kenya (#2761), Washington State University reliance agreement and CDC reliance approval (#6775).

## Results

### Participant enrolment and baseline characteristics

**Figure 3.**
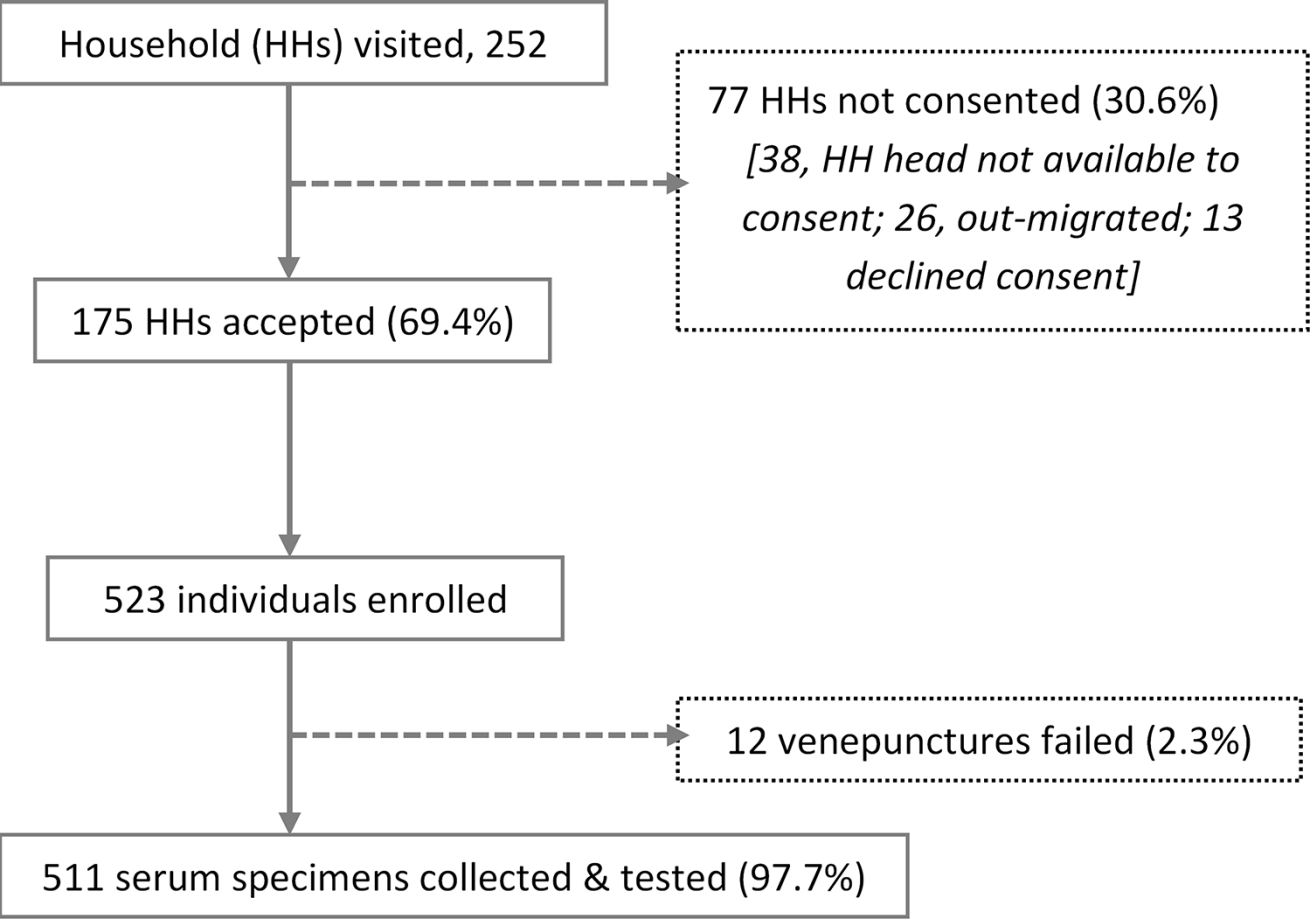
Flow chart showing recruitment process of study household (HHs) and individuals and specimen collection.

Of the 252 randomly selected households, 175 (69.4%) agreed to participate in the survey ( Figure 3). Of the 77 households that did not participate, 38 (49.4%) did not have a household head available for consenting, 26 (33.8%) had moved, and 13 (16.9%) declined participation. Consent was obtained from 523 individuals yielding 511 blood samples; field teams were unable to get a blood specimen from 12 participants. The median number of individuals with a specimen collected per household was 3 (interquartile range, IQR, 2-4; range, 1 – 10).

Of the 511 sampled individuals, 58.5% (299) were female, 23.5% (120) were below the age of 10 and 1.4% (7) were 60 years or older ( Table 1). Males aged 5-9 years and females aged 30-59 years were overrepresented, while both sexes below 5 years and males aged 30 years and above were underrepresented in the surveyed participants relative to the PBIDS general population ( Figure 2).

**Table 1.** Participant characteristics and seroprevalence of severe acute respiratory syndrome coronavirus-2 in Kibera urban informal settlement, Nairobi, Kenya.

Characteristics	Categories	Tested, N	Col ^1^, %	Pos ^2^, n	Crude ^3^, %	wPrev ^4^, %	95% CI ^5^	
Overall	All	511	100.0	222	43.4	43.3	37.4	49.5
Age group, in years	0-4y	41	8.0	3	7.3	7.6	2.4	21.3
	5-9y	79	15.5	24	30.4	32.7	22.9	44.4
	10-19y	150	29.4	62	41.3	41.8	33.0	51.1
	20-29y	84	16.4	46	54.8	60.7	47.8	72.3
	30-39y	82	16.0	47	57.3	48.5	32.5	64.8
	40-49y	42	8.2	23	54.8	53.8	37.8	69.0
	50-59y	26	5.1	13	50.0	51.9	31.6	71.6
	60+y	7	1.4	4	57.1	52.6	13.2	89.0
Sex	Male	212	41.5	83	39.2	43.3	34.4	52.6
	Female	299	58.5	139	46.5	43.4	37.0	50.1
PBIDS zones, areas of residence	1	56	11.0	25	44.6	45.7	27.6	65.1
	2	90	17.6	48	53.3	55.6	39.6	70.6
	3	12	2.3	4	33.3	30.1	11.0	60.0
	4	15	2.9	8	53.3	69.7	47.8	85.3
	5	64	12.5	24	37.5	31.1	21.9	42.1
	6	48	9.4	18	37.5	32.7	17.1	53.2
	7	45	8.8	14	31.1	25.8	8.7	56.0
	8	71	13.9	32	45.1	44.7	31.5	58.8
	9	20	3.9	5	25.0	39.1	17.6	66.0
	10	90	17.6	44	48.9	46.7	35.3	58.4
Relationship to the household head	Self (Household Head)	109	21.3	64	58.7	53.1	41.6	64.2
	Spouse	74	14.5	37	50.0	48.4	35.4	61.7
	Children	292	57.1	104	35.6	37.3	30.4	44.8
	Grandchildren	16	3.1	3	18.8	30.2	8.4	67.2
	Others	20	3.9	14	70.0	63.1	35.1	84.5
Main occupation ^6^	Student	25	4.9	15	60.0	65.0	42.1	82.6
	Unemployed	85	16.6	46	54.1	63.0	50.9	73.7
	Employed - informal	60	11.7	32	53.3	43.4	29.5	58.3
	Business	64	12.5	39	60.9	56.1	39.7	71.3
	Employed - formal	24	4.7	15	62.5	59.8	32.1	82.5
	Healthcare workers	6	1.2	2	33.3	42.9	16.3	74.3
With underlying medical condition	No	473	92.6	204	43.1	42.9	36.7	49.3
	Yes	38	7.4	18	47.4	48.8	29.5	68.5
Specific underlying medical conditions ^6^	Asthma	18	3.5	6	33.3	30.2	11.7	58.7
	Hypertension	12	2.3	6	50.0	47.2	19.4	77.0
	Diabetes	5	1.0	4	80.0	89.0	45.5	98.7
	Others	3	0.6	2	66.7	45.3	6.5	90.8
Highest education Level ^7^	None	7	1.4	2	28.6	21.9	4.4	63.3
	Primary	121	23.7	69	57.0	54.6	42.9	65.9
	Secondary	96	18.8	55	57.3	54.5	41.7	66.8
	Post-secondary	41	8.0	23	56.1	67.2	49.9	80.9
Household size	1	11	2.2	8	72.7	66.0	30.4	89.7
	2	21	4.1	10	47.6	44.6	22.8	68.7
	3	51	10.0	20	39.2	35.8	22.4	51.7
	4	112	21.9	36	32.1	32.1	21.7	44.5
	5	90	17.6	41	45.6	43.2	30.4	57.0
	≥6	222	43.4	104	46.8	48.3	38.6	58.1
Number enrolled per household	1	43	8.4	24	55.8	57.2	39.7	73.0
	2	78	15.3	34	43.6	41.3	28.6	55.2
	3	111	21.7	46	41.4	36.3	25.7	48.4
	4	104	20.4	35	33.7	31.3	19.8	45.5
	5	95	18.6	42	44.2	48.3	35.0	61.8
	≥6	80	15.7	41	51.3	54.7	38.6	69.8

Key: 1, Column percentages, N = 511; 2, Number seropositive; 3, Crude individual seroprevalence; 4, wPrev - Weighted individual seroprevalence accounting for sampling weights and household clustering; 5, 95% Confidence Interval; 6, among those with an underlying medical condition; 7, Data from adults only (≥18 years); PBIDS, the population-based infectious disease surveillance.

### Prevalence of SARS-CoV-2 antibodies

**Figure 4.**
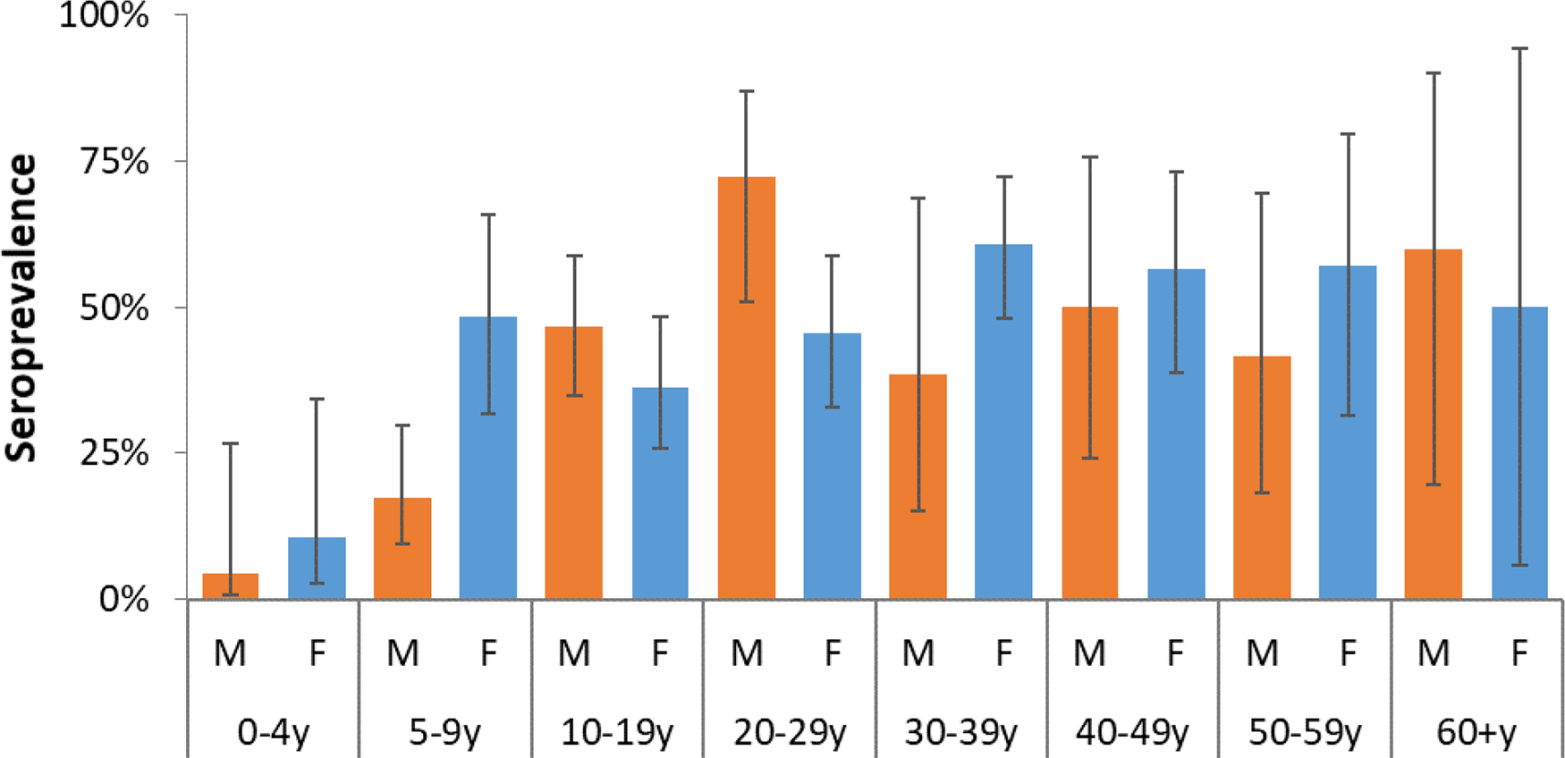
Weighted individual seropositivity of SARS-CoV-2 antibodies stratified by age-sex groups in Kibera urban informal settlement, Nairobi, Kenya.

Of the 511 tested individuals, 222 (43.4%) were seropositive. The overall weighted-seroprevalence was 43.3% (95% CI, 37.4 – 49.5%), with no difference detected between females and males ( Table 1, Figure 4). Seroprevalence increased with age from 7.6% (95% CI, 2.4 – 21.3%) among young children (<5 years), 32.7% (95% CI, 22.9 – 44.4%) among children 5 – 9 years, 41.8% (95% CI, 33.0 – 51.1%) for those 10-19 years, and 54.9% (46.2 – 63.3%) for adults 20 years and above. The elderly (60 years and above) had a seroprevalence of 52.6% (95% CI, 13.2 – 89.0%). The age effect was also observed for seroprevalence estimates by relationships to household head with grandchildren (30.2%; 95% CI, 8.4 – 67.2%) and children (37.3%; 95% CI, 30.4 – 44.8%) registering lower estimates compared to the household head (53.1%; 95% CI, 41.6 – 64.2%) and other adults including spouses (48.4%; 95% CI, 35.4 – 61.7%) and other relatives (63.1%; 95% CI, 35.1 – 84.5%).

The prevalence of SARS-CoV-2 antibodies by area of residence ranged from 25.8% in zone 7 to 69.7% in zone 4. However, the differences in prevalence by area of residence were not statistically significant [Pearson's design-based F statistic = 1.5421, p-value = 0.144]. Participants (≥18 years) with primary, secondary, and post-secondary level of education had similar seroprevalence of 54.6%, 54.5% and 67.2%, respectively. Those with no formal education were few (n = 7) and had a seroprevalence of 21.9% (95% CI, 4.3 – 63.3%). All occupation groups ( Table 1) had a seroprevalence of between 43.3% to 65.0%. Only six health care workers were included in the survey and their seroprevalence was 42.9% (95% CI, 16.3 – 74.3%).

Participants with any underlying medical condition (n = 38) had a seroprevalence of 48.8% (95% CI, 29.5 – 68.5%) which was not statistically different compared with those without, 42.9% (95% CI, 36.7 – 49.3%); (Pearson design-based F statistic = 0.3100, p-value = 0.5784).

**Figure 5.**
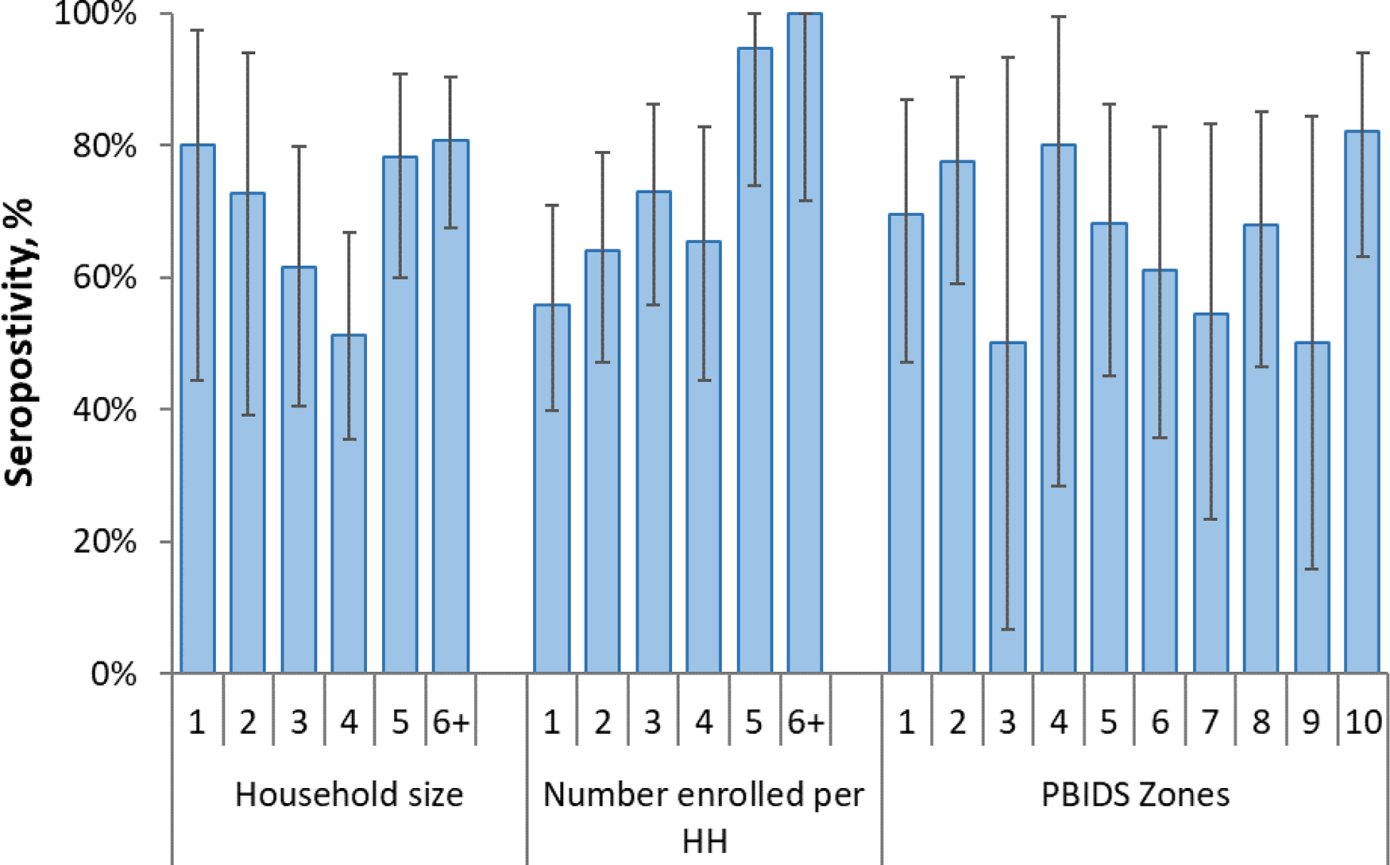
Household (HH) seropositivity (at least one person testing positive in the household) by household size, number of enrolled persons per household and location in the population-based infectious disease surveillance (PBIDS) area as of December 2020. PBIDS zones are areas of residence numbered 1-10.

**Figure 6.**
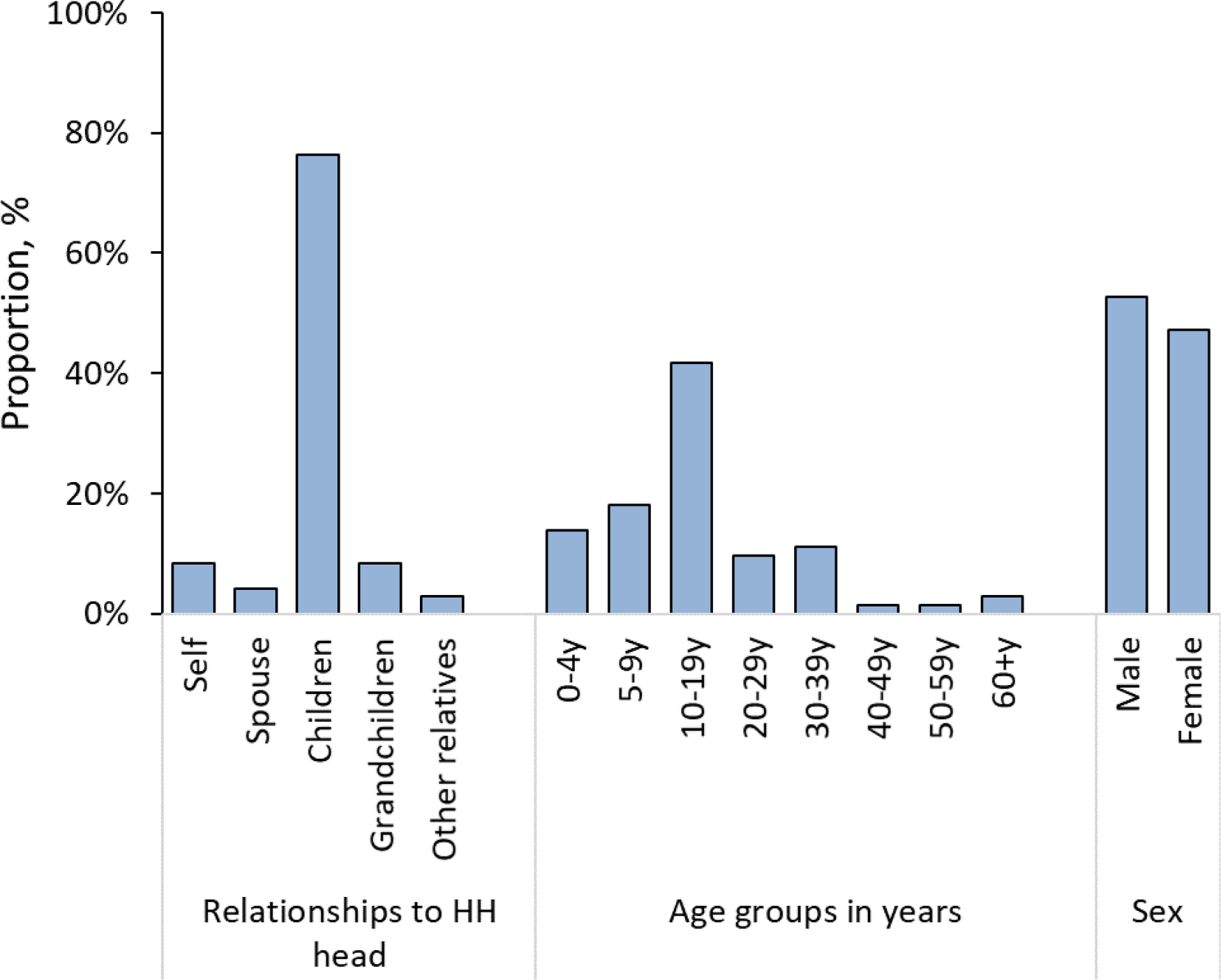
Distribution of 72 seronegative individuals by relationship to household (HH) head, age groups in years and sex from the 98 households with least one seropositive person in Kibera urban informal settlement, Nairobi, Kenya.

Of the households enrolled, 122 (69.7%) had at least one individual with detectable SARS-CoV-2 antibodies ( Table 2). The proportion of households with at least one seropositive individual varied by area of residence ranging from 50.0% in zone 3 and 9 to 82.1% in zone 10, but the differences were not statistically significant ( Figure 5, Table 2). For 132 households with two or more members enrolled, 98 (74.2%) had at least one person seropositive. The median seropositivity within these households with at least one seropositive person (‘exposed’) and two or more participants enrolled was 50.0% (range, 16.7% to 100%). The vast majority (81/98, 82.7%) of these ‘exposed’ households also included one or more seronegative individual(s). The largest proportion of the seronegative household contacts (n = 72) were children of the household head (55, 76.4%), followed by grandchildren of household head (6, 8%), household head (6, 8.3%), spouse (3, 4.2%) and other relatives (2, 2.8%) ( Figure 6).

**Table 2.** Household characteristics and prevalence of households with seropositive individuals in Kibera urban informal settlement, Nairobi, Kenya.

Characteristics	Categories	Tested, N	Col ^1^, %	HH pos ^2^, n	HH prev ^3^, %	95% confidence interval	
Overall	All	175	100.0	122	69.7	62.3	76.4
Household size	1	10	5.7	8	80.0	44.4	97.5
	2	11	6.3	8	72.7	39.0	94.0
	3	26	14.9	16	61.5	40.6	79.8
	4	43	24.6	22	51.2	35.5	66.7
	5	32	18.3	25	78.1	60.0	90.7
	≥6	52	29.7	42	80.8	67.5	90.4
Number enrolled per household	1	43	24.6	24	55.8	39.9	70.9
	2	39	22.3	25	64.1	47.2	78.8
	3	37	21.1	27	73.0	55.9	86.2
	4	26	14.9	17	65.4	44.3	82.8
	5	19	10.9	18	94.7	74.0	99.9
	≥6	11	6.3	11	100	71.5	10
PBIDS zones, areas of residence	1	23	13.1	16	69.6	47.1	86.8
	2	31	17.7	24	77.4	58.9	90.4
	3	4	2.3	2	50.0	6.8	93.2
	4	5	2.9	4	80.0	28.4	99.5
	5	22	12.6	15	68.2	45.1	86.1
	6	18	10.3	11	61.1	35.7	82.7
	7	11	6.3	6	54.5	23.4	83.3
	8	25	14.3	16	68.0	46.5	85.1
	9	8	4.6	4	50.0	15.7	84.3
	10	28	16.0	23	82.1	63.1	93.9

Key: 1, Column percentages, N = 175; 2, Number of households with a seropositive individual; 3, percentage of households with a seropositive individual i.e., household seroprevalence.

### Risk factors for individual seropositivity

Sex, area of residence, relationship to head of household, and underlying medical conditions were not significantly associated with individual seropositivity ( Tables 3 and 4). Individual's age and household size were the independent predictors of seroconversion. Relative to adults aged 20-29 years, young age groups (<20 years) had reduced odds of being seropositive. The odds of being seropositive were similar for older adults (age groups ≥30 years) compared to the reference group, 20-29 years. The odds for being seropositive among the elderly groups (≥60 years) were not different from the referent 20-29 years age group (adjusted odds ratio, aOR, 0.83 (95% CI, 0.19 – 3.64).

**Table 3.** Risk factors for individual seropositivity from univariable logistic regression model in Kibera urban informal settlement, Nairobi, Kenya.

Characteristic	Categories	Odds ratio	95% CI		P-value
Age group	**<5y**	**0.05**	**0.01**	**0.20**	**<0.001**
	**5-9y**	**0.31**	**0.15**	**0.66**	**0.002**
	**10-19y**	**0.46**	**0.24**	**0.89**	**0.021**
	20-29y	Ref			
	30-39y	0.61	0.27	1.39	0.241
	40-49y	0.75	0.33	1.70	0.493
	50-59y	0.70	0.28	1.74	0.439
	≥60y	0.72	0.09	5.60	0.751
Gender	Male	Ref			
	Female	1.01	0.66	1.54	0.978
PBIDS zones, areas of residence	1	Ref			
	2	1.49	0.54	4.11	0.445
	3	0.51	0.12	2.22	0.369
	4	2.73	0.82	9.14	0.103
	5	0.54	0.21	1.34	0.182
	6	0.58	0.18	1.83	0.349
	7	0.41	0.09	1.87	0.25
	8	0.96	0.37	2.52	0.934
	9	0.76	0.20	2.94	0.693
	10	1.04	0.41	2.59	0.938
Relationship to the household head	Household head	Ref			
	Spouse	0.83	0.41	1.68	0.605
	**Children**	**0.53**	**0.32**	**0.87**	**0.013**
	Grandchild	0.38	0.08	1.87	0.235
	Others	1.52	0.45	5.16	0.506
Household size (reported number of household members)	1	Ref			
	2	0.41	0.07	2.48	0.334
	3	0.29	0.06	1.45	0.13
	4	0.24	0.05	1.17	0.078
	5	0.39	0.08	1.91	0.246
	6	0.48	0.10	2.23	0.35
With underlying medical condition	No	Ref			
	Yes	1.27	0.55	2.95	0.578
Number individuals enrolled per household	**1**	**4.11**	**1.18**	**14.3**	**0.026**
	2	1.27	0.45	3.57	0.654
	3	0.68	0.33	1.37	0.278
	4	0.55	0.29	1.03	0.061
	5	0.86	0.44	1.66	0.644
	6	Ref			
Occupation	Student	Ref			
	Unemployed	0.92	0.32	2.67	0.874
	Employed - informal	0.41	0.14	1.19	0.101
	Business owner	0.69	0.23	2.06	0.505
	Employed - formal	0.80	0.20	3.20	0.755
	HCW	0.40	0.08	1.99	0.265
Education level	None	0.23	0.04	1.49	0.124
	Primary	Ref			
	Secondary	1.00	0.52	1.93	0.994
	Post-secondary	1.70	0.73	3.97	0.216

Key: HCW, Health care workers; bold shows statistically significant associations.

**Table 4.** Risk factors for individual seropositivity from multivariable logistic regression model in Kibera urban informal settlement, Nairobi, Kenya.

Characteristic	Categories	Adjusted odds ratio	95% confidence interval		P-value
Age groups in years	**<5y**	**0.06**	**0.02**	**0.21**	**<0.001**
	**5-9y**	**0.36**	**0.17**	**0.76**	**0.008**
	**10-19y**	**0.46**	**0.25**	**0.87**	**0.016**
	20-29y	Ref			
	30-39y	0.79	0.38	1.68	0.544
	40-49y	0.83	0.35	1.95	0.662
	50-59y	0.74	0.28	1.97	0.541
	≥60y	0.83	0.19	3.64	0.802
Sex	Male	Ref			
	Female	1.07	0.69	1.64	0.762
Household size, number of individuals living in the same house	1-2	2.31	0.93	5.74	0.072
	3-4	Ref			
	**≥5**	**1.98**	**1.17**	**3.34**	**0.011**

Key: Ref, reference group; OR were adjusted for age, sex and household size.

Relative to those from medium-sized households (of three and four individuals), participants from large (≥5 persons) households had significantly increased odds of being seropositive, aOR, 1.98 (95% CI, 1.17 – 3.34), while those from small-sized households (≤2 individuals) had increased odds but not statistically significant, aOR 2.31 (95% CI, 0.93 – 5.74).

## Discussion

We report findings from a population-based seroprevalence survey in an urban informal settlement setting in Kenya aligned with WHO's UNITY seroepidemiological protocol. ^18^ An overall seroprevalence of 43.3% in Kibera, the largest urban, densely populated informal settlement in Nairobi, Kenya was observed. A Nairobi-wide serosurvey conducted at the same time utilizing similar methods (specimen collection and testing) reported a lower overall seroprevalence of 32.7% in the County. ^17^ However, the authors noted the seroprevalence in the Nairobi county-wide survey varied across populations with densely populated areas having the highest seroprevalence. The larger Kibera area (known as Kibra subcounty) had a seroprevalence of 42.8%, which corresponds with our finding of 43.3%. Though there are no other published population-based serosurveys in Kenya, estimates from convenient samples of mothers attending antenatal services at Kenyatta National Hospital located in the same administrative area and various cadres of healthcare workers from the same hospital had comparable seroprevalences of 41% and 44%, respectively. ^12, 24^ There are limited serosurveys from informal settlements beyond Kenya but one such study conducted in July 2020 in Mumbai, India found the seroprevalence among slum dwellers to be nearly 3.6 times that of non-slum residents. ^13^ Taken together, the associated challenges for residents of informal settlement to implement mitigation measures such as social distancing, wearing of face masks, and optimal hygiene practices could explain the increased transmission in these populations.

A high level of SARS-CoV-2 exposure in households was recorded with more than two-thirds (69%) of the study households having at least one seropositive member. The seropositivity within households with at least one seropositive person and two or more participants enrolled ranged from 17% to 100% with a median of 50% compared to the overall household seropositivity of 69%. The lower seroprevalence among younger household members suggests transmission outside the household may have played an important role in infection among adults. This finding aligns with observations from the Nairobi county-wide serosurvey and strengthens the argument of increased risk of infection from outside the household, especially among the working populations. ^17^ However, a serosurvey in Singapore showed higher seroprevalence among household contacts compared to work and other social contacts. ^25^ Further, household age structure appeared to play a role with majority of seronegative persons in the exposed households being less than 20 years old. These findings conform to the lower incidence and prevalence of COVID-19 infection found among children in Kenya and other countries. ^26^ Consistent with epidemiological findings of rRT-PCR confirmed SARS-CoV-2 infections, children had a lower cumulative risk of infection than adults. Lower expression of angiotensin converting enzyme 2 in children relative to adults has been considered as one hypothesis for the observed reduced risk. ^27^ Higher prevalence among adolescents 10-19 years compared to younger children below 5 years, also conform with earlier documentation of increasing risk of infection with increasing age among children and adolescents ^28^ which could partly be attributed to increased interaction outside households. Adherence to COVID-19 mitigation measures within the households such as hand hygiene was not assessed in this study but highly unlikely to have reduced the infections rates among younger children given the WASH challenges reported in Kibera. Schools in Kenya had been closed since the confirmation of the first case in March 2020 to the time of this serosurvey, potentially reducing young children's contact to persons living beyond their immediate neighbourhood hence exposure to infectious individuals. The schools have since reopened and a follow up survey would delineate any changes in transmission in the school going children as well as the rest of the population.

Our data show that most of the older persons were in their own business or employment potentially increasing their risk of exposure while on public transport and/or at workplaces. Adherence to mitigation measures may be suboptimal in these settings in Kenya. Although children were not going to school, in the informal settlement with limited indoor space, the anecdotal evidence point to considerable peer interactions as children played outdoors. This is a paradox to disentangle with further investigations when schools open.

Individuals from small (≤2 members) and large (≥5 persons) households had increased odds of being seropositive compared to those from medium-sized (3-4 persons) households. While this observation appears counter intuitive with respect to the role of crowding, most medium-sized households had parents and their children which dovetail well with the lower risk of infection among the young children as discussed earlier. Small-sized households consisted of mainly adults (spouses) who needed to go out for work, while for the larger households, there were more adults, suggesting overcrowding and more adults who needed to go out for work. Sharing of bed space overnight by couples could also partly explain the relatively high risk of infection in the small sized households.

This study had some limitations. First, not all members of the selected households were enrolled, and as shown in Figure 2, the probability of inclusion varied by age and sex. For instance, adult males were underrepresented as they were frequently working at the time of household visits and sampling. This would most likely lead to underestimation of the true population SARS-CoV-2 exposure given the working populations seemed to have higher seroprevalence. However, we have weighted the reported estimates by probabilities of inclusion to generate population level estimates. Second, sample size was limited and possibly inadequate for some of the stratified and regression analyses. Third, the reported seroprevalence was not adjusted for assay performance. Although the Wantai kit was verified in a CDC-supported laboratory, ^17^ sensitivity and specificity estimates from local or similar populations were lacking. The manufacturer's estimates are from a different population to provide meaningful interpretation. The kinetics of antibodies are not fully elucidated, and we may have missed those who were infected several months prior due to waning of detectable antibodies. It would be informative to have serial serosurveys in the seropositive participants to assess the longevity of detectable SARS-CoV-2 antibodies. Finally, seropositivity was not confirmed by a neutralisation or a secondary assay.

In densely populated urban settings where the implementation of mitigation measures – such as case identification and isolation, contact tracing and quarantine, and social distancing – remained very challenging, close to half of the individuals have had aSARS-CoV-2 infection eight months into the COVID-19 pandemic in Kenya. This highlights the importance to prioritize additional mitigation measures, including COVID-19 vaccines, in these crowded, low socioeconomic settings.

## Data availability

The dataset and analyses code are available at Harvard Dataverse. DOI: https://doi.org/10.7910/DVN/LWJH9N. ^29^

This project contains the following underlying data:

- pbids_age_gender_weights.tab
- PBIDS_December_Serosurvey_Dataset_Version_13Aug2021.tab

Data are available under the terms of the Creative Commons Zero “No rights reserved” data waiver (CC0 1.0 Public domain dedication).

wAccess to the dataset is restricted as it contains sensitive participant identifying information. Accompanying documentation is available under open access. For more detailed information beyond the metadata and documentation provided, there is a process of managed access requiring submission of a request, detailing the intended use, for consideration by our Data Governance Committee. Please contact the Data Governance Committee via this email address - gbigogo@kemricdc.org.

### Disclaimer

The findings and conclusions in this study are those of the authors and do not necessarily represent the official position of the US National Institutes of Health, KEMRI, or U.S. Centers for Disease Control and Prevention.
