# Supplementary material for: Seroprevalence and risk factors of SARS-CoV-2 infection in an urban informal settlement in Nairobi, Kenya, December 2020
Source: F1000Res. 2022 Apr 11;10:853. Originally published 2021 Aug 26. [Version 2] doi: 10.12688/f1000research.72914.2 (PMC9065925; doi:10.12688/f1000research.72914.2)
Supplement: Peer review history

# Peer review: Reviewer response for version 2

**DOI:** 10.5256/f1000research.125825.r134543

**Published:** 2022-05-03

**Authors:** Sarah R. Haile

**Affiliations:** 1 Epidemiology, Biostatistics and Prevention Institute (EBPI), University of Zurich, Zurich, Switzerland

No further comments

Is the work clearly and accurately presented and does it cite the current literature?

Yes

If applicable, is the statistical analysis and its interpretation appropriate?

Partly

Are all the source data underlying the results available to ensure full reproducibility?

Yes

Is the study design appropriate and is the work technically sound?

Yes

Are the conclusions drawn adequately supported by the results?

Yes

Are sufficient details of methods and analysis provided to allow replication by others?

Yes

Reviewer Expertise:

I am biostatistician with experience in both clinical trials and epidemiological (incl. observational) data. I work in a variety of different subject areas, including a study of covid seroprevalence in Swiss children.

I confirm that I have read this submission and believe that I have an appropriate level of expertise to confirm that it is of an acceptable scientific standard.

# Peer review: Reviewer response for version 1

**DOI:** 10.5256/f1000research.76527.r126232

**Published:** 2022-03-28

**Authors:** Tatjana Vilibic-Cavlek

**Affiliations:** 1 Department of Virology, Croatian Institute of Public Health, School of Medicine, University of Zagreb, Zagreb, Croatia

I have read with great interest the manuscript entitled "Seroprevalence and risk factors of SARS-CoV-2 infection in an urban informal settlement in Nairobi, Kenya, December 2020". The manuscript is generally well written and the results are clearly presented. Please see some minor comments regarding the methodology below.

**Methods:**

- Data and specimen collection
  Page 5, line 6: please correct "Serum samples were extracted from the whole blood ..."
- Serological testing
  Page 5, lines 1-3: please add characteristics of the ELISA test used for detection of SARS-CoV-2 antibodies (sensitivity, specificity).

In my opinion, one of the main limitation of the study is that confirmatory virus neutralization test has not been performed. However, the authors stated this limitation in the discussion section.

Is the work clearly and accurately presented and does it cite the current literature?

Yes

If applicable, is the statistical analysis and its interpretation appropriate?

Yes

Are all the source data underlying the results available to ensure full reproducibility?

Yes

Is the study design appropriate and is the work technically sound?

Yes

Are the conclusions drawn adequately supported by the results?

Yes

Are sufficient details of methods and analysis provided to allow replication by others?

Yes

Reviewer Expertise:

I am a Medical Microbiology specialist. I work at the Department of Virology, and my areas of research include emerging and re-emerging viral zoonoses, hepatitis viruses and TORCH infections.  I have participated in  seroepidemiological studies of COVID-19 in different population groups in Croatia.

I confirm that I have read this submission and believe that I have an appropriate level of expertise to confirm that it is of an acceptable scientific standard.

# Response

**Published:** 2022-04-07

**Authors:** Patrick K Munywoki

**Affiliations:** CDC Kenya, Kenya

Thank you for the review and the minor comments raised. 

 A detailed response to the review comments is provided below:

 1. Data and specimen collection: Page 5, line 6: please correct "Serum samples were extracted from the whole blood ..."

*We have corrected the statement as advised. Thank you*

 2. Serological testing, Page 5, lines 1-3: please add characteristics of the ELISA test used for detection of SARS-CoV-2 antibodies (sensitivity, specificity)

*We have added the reported sensitivity and specificity of the serological assay used* *(* *https://www.ystwt.cn/wp-content/uploads/2020/05/Brochure-Wantai-SARS-CoV-2-Ab-ELISA.pdf* *).*

 3. In my opinion, one of the main limitation of the study is that confirmatory virus neutralization test has not been performed. However, the authors stated this limitation in the discussion section.

*We acknowledge the limitation on lack of confirmatory virus neutralization test and point that out in the study limitation section. Thank you*

# Peer review: Reviewer response for version 1

**DOI:** 10.5256/f1000research.76527.r92857

**Published:** 2021-09-01

**Authors:** Sarah R. Haile

**Affiliations:** 1 Epidemiology, Biostatistics and Prevention Institute (EBPI), University of Zurich, Zurich, Switzerland

Thank you for the opportunity to review this well-written manuscript. I've seen similar work on seroprevalence in other populations recently, and such studies are certainly complicated to perform and analyse.

 My main question is what are the test performance characteristics of the antibody test used (sensitivity, specificity)? If I have missed them in a citation, I apologize but would ask that they be restated here for completeness.

 The statistical analysis took care of many factors of the study design (household clustering and population weights, for example). Would it nevertheless be possible to explicitly account for test sensitivity and specificity, as performed for example in this study ^1^ (see also the supplementary material for full model write-up and link to GitHub repository)?

Is the work clearly and accurately presented and does it cite the current literature?

Yes

If applicable, is the statistical analysis and its interpretation appropriate?

Partly

Are all the source data underlying the results available to ensure full reproducibility?

Yes

Is the study design appropriate and is the work technically sound?

Yes

Are the conclusions drawn adequately supported by the results?

Yes

Are sufficient details of methods and analysis provided to allow replication by others?

Yes

Reviewer Expertise:

I am biostatistician with experience in both clinical trials and epidemiological (incl. observational) data. I work in a variety of different subject areas, including a study of covid seroprevalence in Swiss children.

I confirm that I have read this submission and believe that I have an appropriate level of expertise to confirm that it is of an acceptable scientific standard, however I have significant reservations, as outlined above.

## References

[1] : Seroprevalence of anti-SARS-CoV-2 IgG antibodies in Geneva, Switzerland (SEROCoV-POP): a population-based study. *The Lancet* .2020;396(10247) : 10.1016/S0140-6736(20)31304-0 313-319 10.1016/S0140-6736(20)31304-0 PMC728956432534626

# Response

**Published:** 2022-04-07

**Authors:** Patrick K Munywoki

**Affiliations:** CDC Kenya, Kenya

Thank you for the review and the very useful comments.

 We tested for total immunoglobulins (IgM and IgG) antibodies to the SARS-CoV-2 spike protein using the Wantai SARS-CoV-2 two-step antigen sandwich enzyme immunoassay kit (Catalogue number, WS-1096; Wantai Biological Pharmacy Enterprise Ltd, Beijing, China) whose published sensitivity and specificity is 94.4% and 100% respectively from populations in China ( https://www.ystwt.cn/wp-content/uploads/2020/05/Brochure-Wantai-SARS-CoV-2-Ab-ELISA.pdf). However, local or data from similar populations is lacking on the test performance hence the reported seroprevalence did not account for the sensitivity and specificity. We have made this decision explicit in the revised manuscript including in the discussion as a limitation. 
